# Comprehensive analysis of m^6^A/m^5^C/m^1^A-related gene expression, immune infiltration, and sensitivity of antineoplastic drugs in glioma

**DOI:** 10.3389/fimmu.2022.955848

**Published:** 2022-09-20

**Authors:** Kai Zhao, Wenhu Li, Yongtao Yang, Xinyue Hu, Ying Dai, Minhao Huang, Ji Luo, Kui Zhang, Ninghui Zhao

**Affiliations:** ^1^Neurosurgery Department, the Second Affiliated Hospital of Kunming Medical University, Kunming, China; ^2^Cerebrovascular Disease Department, the Second Affiliated Hospital of Kunming Medical University, Kunming, China; ^3^Department of Clinical Laboratory, Kunming First People’s Hospital, Kunming Medical University, Kunming, China

**Keywords:** glioma, RNA methylation modification, N6-adenylate methylation (m^6^A), N1-adenylate methylation (m^1^A), cytosine hydroxylation (m^5^C), tumor immune microenvironment

## Abstract

This research aims to develop a prognostic glioma marker based on m^6^A/m^5^C/m^1^A genes and investigate the potential role in the tumor immune microenvironment. Data for patients with glioma were downloaded from The Cancer Genome Atlas (TCGA) and Chinese Glioma Genome Atlas (CGGA). The expression of genes related to m^6^A/m^5^C/m^1^A was compared for normal and glioma groups. Gene Ontology and Kyoto Encyclopedia of Genes and Gene enrichment analysis of differentially expressed genes were conducted. Consistent clustering analysis was performed to obtain glioma subtypes and complete the survival analysis and immune analysis. Based on TCGA, Lasso regression analysis was used to obtain a prognostic model, and the CGGA database was used to validate the model. The model-based risk scores and the hub genes with the immune microenvironment, clinical features, and antitumor drug susceptibility were investigated. The clinical glioma tissues were collected to verify the expression of hub genes *via* immunohistochemistry. Twenty genes were differentially expressed, Consensus cluster analysis identified two molecular clusters. Overall survival was significantly higher in cluster 2 than in cluster 1. Immunological analysis revealed statistically significant differences in 26 immune cells and 17 immune functions between the two clusters. Enrichment analysis detected multiple meaningful pathways. We constructed a prognostic model that consists of *WTAP*, *TRMT6*, *DNMT1*, and *DNMT3B*. The high-risk and low-risk groups affected the survival prognosis and immune infiltration, which were related to grade, gender, age, and survival status. The prognostic value of the model was validated using another independent cohort CGGA. Clinical correlation and immune analysis revealed that four hub genes were associated with tumor grade, immune cells, and antitumor drug sensitivity, and *WTAP* was significantly associated with microsatellite instability(MSI). Immunohistochemistry confirmed the high expression of WTAP, DNMT1, and DNMT3B in tumor tissue, but the low expression of TRMT6. This study established a strong prognostic marker based on m^6^A/m^5^C/m^1^A methylation regulators, which can accurately predict the prognosis of patients with gliomas. m^6^A/m^5^C/m^1^A modification mode plays an important role in the tumor microenvironment, can provide valuable information for anti-tumor immunotherapy, and have a profound impact on the clinical characteristics.

## 1 Introduction

Glioma is the most common primary malignant tumor of the central nervous system (CNS) and originates from astrocytes, oligodendrocytes, and ependymal glial cells. The annual incidence rate of glioma is approximately 6/100,000, and the prevalence rate in males is 1.6 times higher than that in females (1). Because Roman numerals II and III are easily confused, cIMPACT-NOW now recommends using Arabic numerals to grade gliomas, in which low-grade gliomas (LGG) include CNS WHO grades 1–2, while high-grade gliomas (HGG) contain CNS WHO grades 3–4 (2). The median overall survival time of LGG and HGG is 78.1 and 14.4 months, respectively (3). Glioblastoma (GBM) is the most common type of glioma, accounting for 57% of all gliomas and 48% of all primary malignant tumors of the CNS. It is one of the deadliest and most common malignant solid tumors. The therapeutic effect of total surgical resection combined with postoperative concurrent chemoradiotherapy on GBM is still insufficient. Malignant glioma has a five-year survival rate of only 4–29%, the survival time is approximately one year, and there is no cure (4). Exploration of effective biological targets, understanding the complex pathogenesis and molecular mechanism of glioma, and developing effective treatment strategies are urgently needed.

RNA modification is a critical step in epigenetics for regulating post-transcriptional gene expression, and it has emerged as the most important post-transcriptional regulator of gene expression programs, with RNA base methylation modification being the most common. The most prevalent types of RNA modification are N6-adenylate methylation (m^6^A), N1-adenylate methylation (m^1^A), and cytosine hydroxylation (m^5^C) (5). m^6^A, commonly known as N6- methyladenosine, is the methylated sixth nitrogen atom of adenine. In RNA transcripts, m^6^A is the most common chemical alteration. Several m^6^A components (writers, readers, and erasers) have been linked to cancer and proposed as prospective therapeutic targets (6, 7). m^1^A is another key methyltransferase-catalyzed post-transcriptional RNA modification. Unlike m^6^A, adenylate transformed by m^1^A is methylated at the N1 location. In multiple cancer cell lines, the m^1^A regulator demethylated tRNAs and created short RNAs derived from tRNAs to enhance cancer cell growth (8). The methylation of the fifth C atom of RNA cytosine is known as m^5^C. m^5^C regulates the stability, expression, and translation of mRNA, which is critical for cancer cell proliferation and metastasis as well as tumor stem cell development (9–11). Lin et al. (12) explored the differentially expressed m^6^A regulatory genes in gliomas through the Cancer Genome Atlas (TCGA) and found that PDPN and TIMP1 may be potential biomarkers of glioma prognosis. Li (13) showed that m^5^C-related genes could predict the survival rate and prognosis of low-grade gliomas, in which the expression of *NSUN3*, *TET2*, *TRDMT1*, *ALYREF*, *DNMT3B*, *DNMT1*, *NOP*2, *NSUN2* were upregulated, and *DNMT3A* mutation was the most common type. However, these studies have some limitations, such as having a single dataset, small sample size, incomplete analysis, and no combination of clinical characteristics and immune correlation analysis. In addition, there is no bioinformatics study of m^1^A in gliomas. Therefore, to build a more accurate prognostic model and identify the potential prognostic biomarkers, it is necessary to conduct an in-depth analysis of m^6^A/m^5^C/m^1^A-related genes in gliomas.

This study used TCGA and CGGA databases to acquire RNA sequencing data and clinical information from glioma patients to explore the potential role of m^6^A/m^5^C/m^1^A-associated genes in glioma. After the difference analysis of m^6^A/m^5^C/m^1^A-related genes *via* consensus cluster analysis, risk models were created to better predict the prognosis of patients with glioma. We used the glioma-related data from the CGGA database to test the model’s accuracy. In addition, we investigated the role of m^6^A/m^5^C/m^1^A-associated genes in immune infiltrating cells, immunological function, clinical characteristics, and chemosensitivity in glioma, to determine potentially effective biomarkers.

## 2 Materials and methods

### 2.1 Data collection and genes related to m^6^A/m^5^C/m^1^A

The GBM and LGG samples, including 5 control samples, 500 LGG samples, and 145 GBM samples, were retrieved from TCGA, and clinical data of patients, including sex, living situation, and follow-up period, were obtained. The GBM and LGG datasets were integrated, tumor patients with travel follow-up data were screened (**Table 1**), and patient microsatellite instability data were obtained (14). The CCGA (15) data, DataSet ID: mRNAseq 693, which includes 693 patients with glioma, were downloaded to obtain the matched clinical data, such as living conditions and follow-up period, for verifying the TCGA dataset. m^6^A has identified 23 genes. m^5^C/m^1^A-related genes are mainly derived from the literature, but some genes are not detected in the two data sets of TCGA or CGGA. Finally, there are 41 genes associated with m^6^A/m^5^C/m^1^A were collected (**Supplement Material 1**).

**Table 1:** Glioma patients with clinical data in TCGA.

Category		Number
Overall		638
OS.Dead (%)		236 (37.0)
Grade (%)	G2	207 (32.4)
	G3	232 (36.4)
	G4	143 (22.4)
	Unknown	56 (8.8)
Gender.Male (%)		369 (57.8)
Age ≤ 60 (%)		556 (87.1)

OS, Overall Survival.

### 2.2 Bioinformatic analysis

Deseq2 package and rank sum test were used to compare the differences in genes related to m^6^A/m^5^C/m^1^A between the normal samples and glioma tumor samples in TCGA and display the differences with a box plot, P<0.05 represents a significant difference. The clusterProfiler R software package (16) was used to explore the biological processes, molecular function,cellular components and the Kyoto Encyclopedia of Genes and Genomes (KEGG) of the m^6^A/m^5^C/m^1^A related differentially expressed genes,the results with P<0.05 & q<0.05 was considered to be statistically significant. The STRING database (17) is a database of the interactions between known and predicted proteins. The protein-protein interaction network between m^6^A/m^5^C/m^1^A-related genes is constructed in the STRING database. The coefficient was set as 0.7, and different colors represent log2FC values. The protein-protein interaction results were derived from the STRING database and further visualized using Cytoscape (18). The correlation network diagram and chromosome distribution diagram of the m^6^A/m^5^C/m^1^A genes were created.

The differentially expressed genes were analyzed *via* univariate Cox regression analysis, and then the m^6^A/m^5^C/m^1^A differentially expressed genes with prognostic values were detected. The “ConsensusClusterPlus” R package (19) was used for consistent cluster analysis to better distinguish the different clusters of gliomas. The number of clusters was set from 2 to 5, 80% of the total samples collected were repeated 100 times using clusterAlg =”pam”, distance=”euclidean”. To study the differences in biological processes among subtypes, gene set enrichment analysis (GSEA) was used, and the results with P <0.05 were considered significantly enriched. The differentially expressed genes of m^6^A/m^5^C/m^1^A with prognostic values among clusters and the survival of different clusters were analyzed. The immune function gene set was downloaded from the marker (**Supplement Material 2**) of 28 types of immune cells and the Immport database (https://www.immport.org/resources) (**Supplement Material 3**). The single-sample GSEA (ssGSEA) of TCGA glioma samples was carried out using the gene set variation analysis (GSVA) package (20). The composition and abundance of 28 types of immune cells in glioma samples were estimated, and the differences between different subtypes of immune cells and immune function were compared.

In TCGA, the correlation between the expression of the differentially expressed gene and overall survival (OS) was calculated using univariate Cox regression analysis, and the genes with P <0.1 were retained. Then, the lasso regression was used to eliminate multiple collinearity and screen the variables for univariate Cox regression analysis. To obtain more accurate independent prognostic factors (prognostic characteristic genes), a prognostic model was established by using multivariate Cox regression analysis. The risk score formula was established as follows: risk score = (exp-Gene1*coef-Gene1) + (exp-Gene2*coef-Gene2) +… + (exp-Gene*coef-Gene). According to the given risk score, the patients were divided into the high-risk group and the low-risk group. Kaplan-Meier analysis and logarithmic rank test were performed using a survival package to analyze the OS of the test set. In addition, the time-dependent subject operating characteristic (ROC) curve was used to evaluate survival prediction, the timeROC package (21) was used to calculate the area under the curve (AUC) to measure prognosis or prediction accuracy, and the model was verified using the CGGA database. The differences in immune cells and immune function between the high- and low-risk groups were analyzed. Finally, the relationship between the risk score and tumor grade, sex, survival status, and age was determined.

To study the key genes in the model, we first mapped the distribution of these genes in high- and low-risk groups and analyzed their relationship with tumor grade, MSI, and immune cells. The drug sensitivity was analyzed using the CellMiner database (https://discover.nci.nih.gov/cellminer/). The correlation between the expression of key genes and compound sensitivity was calculated by using Pearson’s correlation coefficient. The values with P <0.05 were considered statistically significant.

### 2.3 Immunohistochemical verification and ethics approval statement

Fresh glioma tissues and paracancerous tissues were collected, and the expression of hub genes was verified by using an immunohistochemical experiment. Xylene and anhydrous ethanol were added to the tissue-fixed embedded slices, which were alcohol-soaked and then washed with distilled water. After the antigen was repaired, it was heated in the microwave oven and cleaned with phosphate buffered saline after cooling. Bovine serum albumin was dripped into the submerged tissue, and then WTAP (Abbkine), TRMT6 (Cusabio), DNMT1 (Abcam) and DNMT3B (Abbkine) were added. The tissue was incubated overnight in a wet box at 4°C, and DAB chromogenic solution was added. The tissue was then restained and differentiated with hematoxylin. The tissue slices were dehydrated and cleared to make them transparent, and the neutral gum was sealed after drying. Under a microscope, the images were taken: negative without coloring, weak positive light yellow, medium positive brown, and strong positive brown. In this clinical study, the patients and their families have been informed about the project and they have signed written consent. The project has been approved by the Clinical Ethics Committee of the Second Affiliated Hospital of Kunming Medical University (Code : PJ-2021-106).

### 2.4 Statistical analysis

All calculations and statistical analysis were carried out using R software (https://www.r-project.org/Magiversion4.0.2). For the comparison of two groups of continuous variables, the differences between variables were analyzed by using the Mann-Whitney U test (Wilcoxon rank-sum test). For the comparison of more than two groups of continuous variables, the differences between variables were analyzed *via* the Kruskal-Wallis test. All the statistical p values were bilateral, and the results with P <0.05 were regarded as statistically significant.

## 3 Results

### 3.1 Technical flow chart of this study 

**Figure 1:**
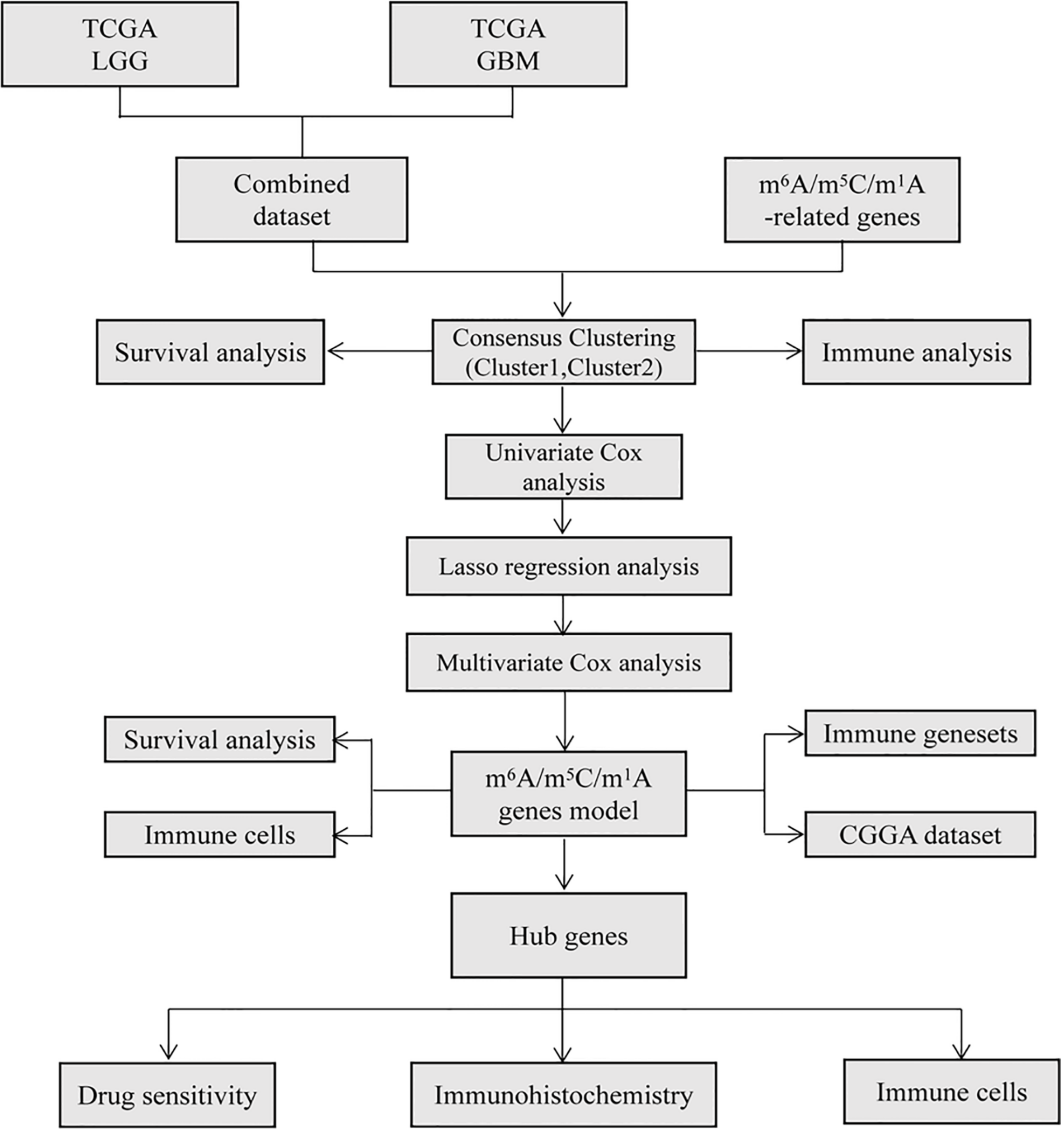
Flow chart in the current study. TCGA database was used to extract and integrate glioma data, and the differences of m^6^A/m^5^C/m^1^A related genes were analyzed. Based on these differentially expressed genes, different glioma clusters were obtained by consistent cluster analysis, and the survival and immunity of different clusters were analyzed. The prognostic model was constructed by Cox and Lasso regression, the risk score was calculated, the survival and immunity between high and low risk groups were compared, and the accuracy of the model was verified by CGGA database. Finally, the correlation between hub genes and immune cells and the sensitivity of drugs were analyzed and verified by immunohistochemistry.

**Figure 1** shows the technical flow chart of our study.

#### 3.1.1 Differentially expressed gene analysis and correlation of m^6^A/m^5^C/m^1^A-related genes

**Figure 2:**
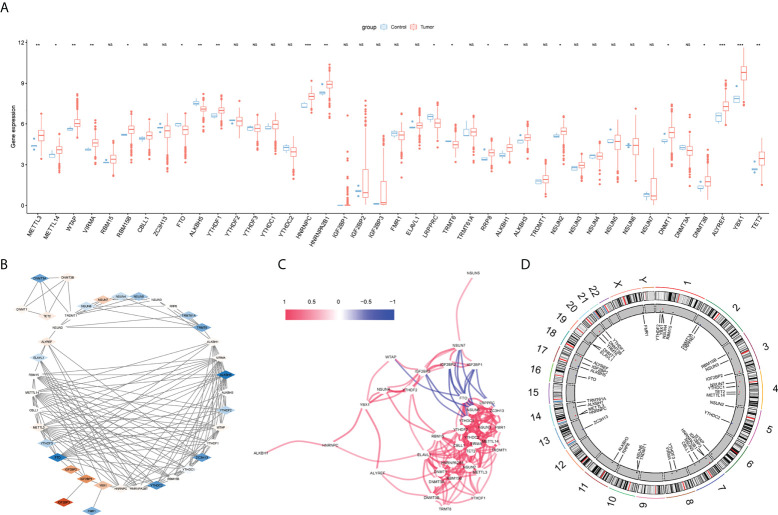
m^6^A/m^5^C/m^1^A related genes analysis. **(A)** The difference expression of m^6^A/m^5^C/m^1^A related genes between glioma and control samples, with red representing tumor group and blue representing normal group. **(B)** Protein-protein interaction diagram, different colors represent log2FC values (obtained by comparing normal group and tumor group with Deseq2 package), orange represents log2FC >0, blue represents log2FC < 0, and the darker the color, the larger |log2FC|. **(C)** Correlation network diagram, red line represents positive correlation and blue represents negative correlation; chromosome map shows the distribution of genes related to m^6^A/m^5^C/m^1^A on chromosomes **(D)**. (NS:no significant) (*P < 0.05, **P < 0.01, ***P < 0.001).

In the difference analysis of 41 m^6^A/m^5^C/m^1^A-related genes between glioma and normal samples, 20 genes (*METTL3*, *METTL14*, *WTAP*, *VIRMA*, *RBM15B*, *FTO*, *ALKBH5*, *YTHDF1*, *HNRNPC*, *HNRNPA2B1*, *LRPPRC*, *TRMT6*, *RRP8*, *ALKBH1*, *NSUN2*, *DNMT1*, *DNMT3B*, *ALYREF*, *YBX1,TET2*) showed significant differences (**Figure 2A**, **Table 2**). The interaction network map of m^6^A/m^5^C/m^1^A-related genes was obtained by using the STRING database and visualized using Cytoscape software(Version 3.7.1). Different colors in **Figure 2** represent the log2FC values obtained *via* difference analysis: orange represents log2FC >0, blue represents log2FC <0, and the darker the color, the larger the |log2FC| value (**Figure 2B**). We analyzed the correlations between the m^6^A/m^5^C/m^1^A-related genes, drew the correlation network diagram, and showed the genes with an absolute value of the correlation coefficient greater than 0.4 (**Figure 2C**). A chromosome map (**Figure 2D**) was drawn to show the distribution of m^6^A/m^5^C/m^1^A-related genes on chromosomes.

**Table 2:** Differentially expressed analysis of m^6^A/m^5^C/m^1^A-related genes.

Gene	type	P
METTL3	m^6^A	0.0044
METTL14	m^6^A	0.0265
WTAP	m^6^A	0.0059
VIRMA	m^6^A	0.0047
RBM15B	m^6^A	0.0104
FTO	m^6^A	0.0107
ALKBH5	m^6^A	0.0011
YTHDF1	m^6^A	0.0035
HNRNPC	m^6^A	0.0004
HNRNPA2B1	m^6^A	0.0097
LRPPRC	m^6^A	0.0296
TRMT6	m^1^A	0.0473
RRP8	m^1^A	0.0429
ALKBH1	m^1^A	0.0038
NSUN2	m^5^C	0.0249
DNMT1	m^5^C	0.0175
DNMT3B	m^5^C	0.0294
ALYREF	m^5^C	0.0009
YBX1	m^5^C	0.0002
TET2	m^5^C	0.009

### 3.2 GO and KEGG enrichment analysis

**Figure 3:**
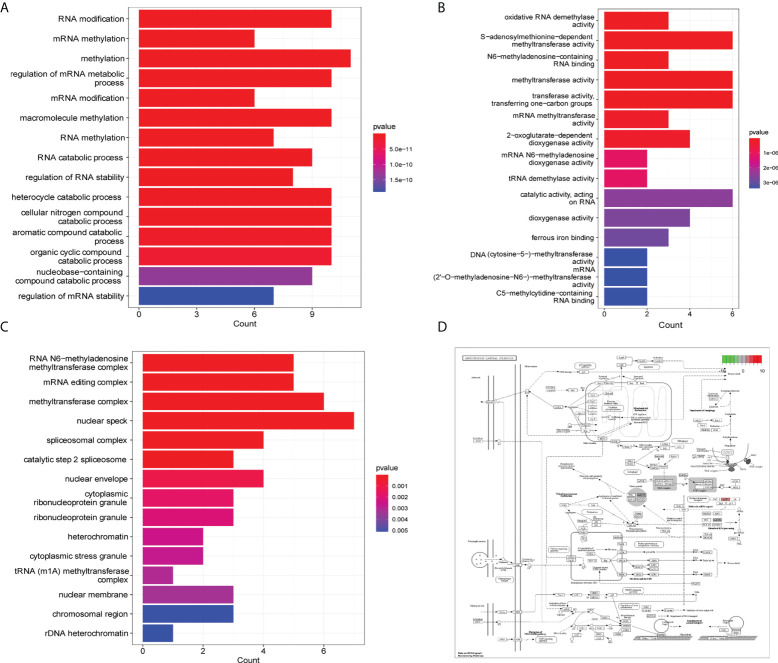
GO and KEGG enrichment analysis. **(A)** GO enrichment analysis histogram (biological process), the abscissa is the number of genes, the ordinate is GO term, and the color indicates padj. **(B)** Histogram of enrichment analysis (molecular function), the abscissa is gene number, the ordinate is GO term, and the color indicates padj. **(C)** Enrichment analysis histogram (cell components), the abscissa is the number of genes, the ordinate is the GO term, and the color indicates padj. **(D)** Path diagram of hsa05014.

We analyzed the biological processes, molecular function, cell components, and related pathways of the differentially expressed m^6^A/m^5^C/m^1^A-related genes. They affect biological processes such as RNA modification, mRNA methylation, methylation, regulation of mRNA metabolic processes (**Figure 3A**), oxidative RNA demethylase activity, S-adenosylmethionine-dependent methyltransferase activity, N6-methyl adenine-containing RNA binding, methyltransferase activity and other molecular functions (**Figure 3B**), RNA N6-methyladenosine methyltransferase complex, mRNA editing complex, methyltransferase complex, cell components such as methyltransferase complex (**Figure 3C**: **Supplement Material 4**), and pathways such as cysteine and methionine metabolism, spliceosome, and microRNAs in cancer (**Table 3**). We also showed the hsa05014 pathway associated with cancer (**Figure 3D**).

**Table 3:** KEGG enrichment analysis of differentially expressed m^6^A/m^5^C/m^1^A-related genes.

ID	Description	P	Count
hsa00270	Cysteine and methionine metabolism	0.0003	2
hsa03040	Spliceosome	0.0003	2
hsa05206	MicroRNAs in cancer	0.0134	2
hsa05014	Amyotrophic lateral sclerosis	0.0183	2
hsa03015	mRNA surveillance pathway	0.0583	1
hsa03013	Nucleocytoplasmic transport	0.0648	1
hsa05168	Herpes simplex virus 1 infection	0.2700	1

### 3.3 Glioma cluster analysis

**Figure 4:**
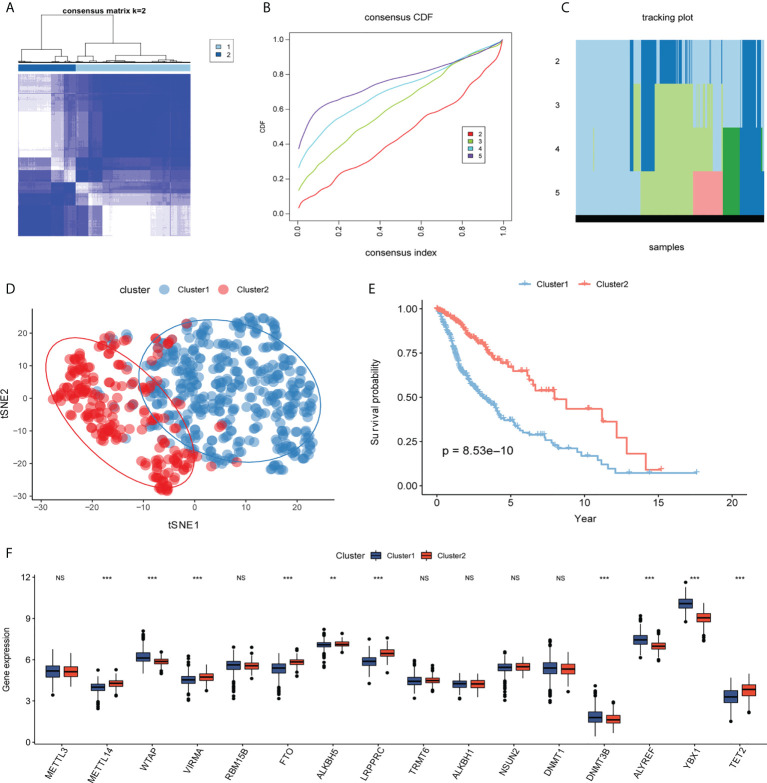
Molecular typing. **(A–C)** Consistent clustering of differentially expressed m^6^A/m^5^C/m^1^A-related genes (k=2–5). **(D)** According to t-distributed stochastic neighbor embedding (t-SNE) analysis, there is a good distinction between different cluster samples, with blue representing cluster 1 and red representing cluster2. **(E)** Kaplan–Meier curve between different clusters, blue represents cluster1, and red represents cluster2. **(F)** The box plot shows the expression levels of genes related to m^6^A/m^5^C/m^1^A among different clusters, with blue representing cluster1 and red representing cluster2. (NS:no significant) (**P < 0.01, ***P < 0.001).

Based on the glioma samples from TCGA, 20 differentially expressed m^6^A/m^5^C/m^1^A-related genes were analyzed using the univariate Cox regression analysis, and 16 genes were found to have prognostic significance (P< 0.1, **Supplement Material 5**). Cluster analysis identified distinct glioma subtypes (k=2–5) based on these 16 genes, and k=2 was chosen to divide gliomas into two clusters (**Figures 4A-C**). t-Stochastic neighbor embedding (tSNE) showed the differentiation between different subcategories (**Figure 4D**). The overall survival rate of patients in cluster 2 was significantly higher than that in cluster 1 (**Figure 4E**). We used a box chart to show the expression levels of 16 genes between different clusters. Ten genes (*METTL14*, *WTAP*, *VIRMA*, *FTO*, *ALKBH*, *LRPPRC*, *DNMT3B*, *ALYREF*, *YBX1*, *TET2*) were differentially expressed (**Figure 4F**).

### 3.4 GSEA and immune correlation analysis among different clusters

**Figure 5:**
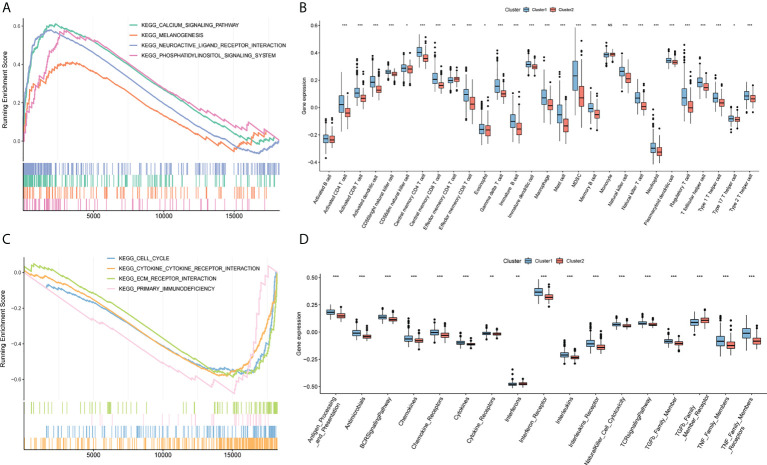
GSEA analysis. **(A)** Calcium signaling pathway, melanogenesis, neuroactive ligand-receptor interaction, and phosphoinositol signaling system are significantly enriched in cluster2. **(B)** Box plot of the difference of immune infiltration abundance between cluster1 and cluster2. The horizontal axis represents immune cells, the vertical axis represents immune cell infiltration abundance, blue represents cluster1, and red represents cluster2. **(C)** Cell cycle, primary immunodeficiency, cytokine receptor interaction, and extracellular matrix-receptor interaction are significantly enriched in cluster1. **(D)** Box plot of cluster1 and cluster2 and immune function set score. The horizontal axis represents the immune function set, the vertical axis represents the immune function set score, blue represents cluster1, and red represents cluster2. (NS:no significant) (*P < 0.05, **P < 0.01, ***P < 0.001).

To further analyze the differences in pathways between different clusters, GSEA was used to show that the calcium signaling pathway, melanogenesis, neuroactive ligand-receptor interaction, and phosphatidylinositol signaling system were significantly enriched in cluster 2 (**Figure 5A**). Cell cycle, primary immunodeficiency, cytokine receptor interaction, and extracellular matrix-receptor interaction are significantly enriched in cluster 1 (**Figure 5C**; **Supplement Material 6**). 28 types of cells in clusters 1 and 2 showed statistical differences except for activated B cells and monocytes, and 17 immune function gene set scores showed significant differences between the two groups with ssGSEA to calculate the immune cell infiltration and immune function gene set score of glioma (**Figures 5B, D****)**.

### 3.5 Establishment of the prognostic model

**Figure 6:**
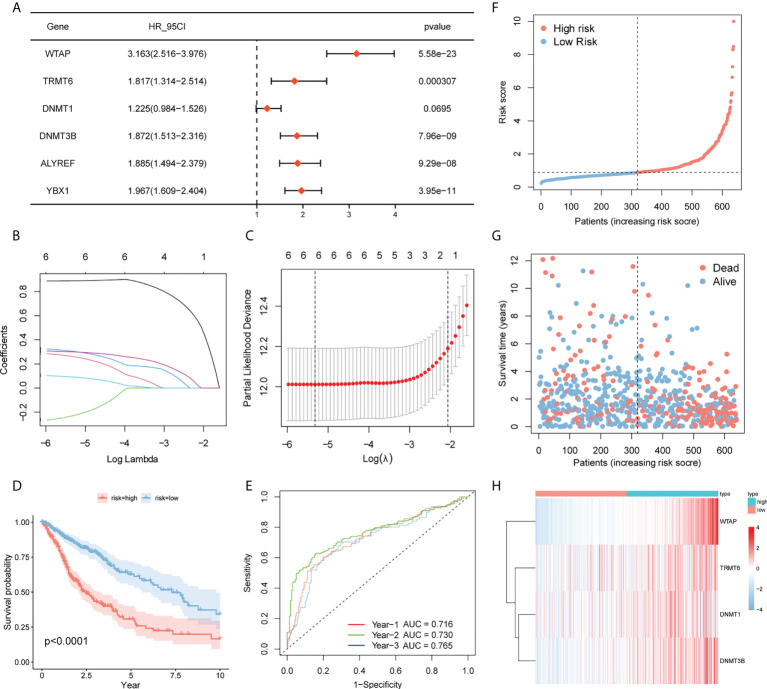
Prognostic model. **(A)** Screening of genes with P <0.1 by univariate Cox regression analysis. **(BC)** Lasso–Cox regression to screen the related genes of m^6^A/m^5^C/m^1^A. **(D)** Survival curves of high- and low-risk groups in TCGA. **(E)** Time-dependent ROC curve for prognosis based on risk score. **(F–H)** Risk trigram showing risk distribution of patients and expression of key genes in different subgroups.

As a starting point, we performed a univariate Cox regression analysis on TCGA and CGGA data to seek characteristics linked to OS in patients with glioma and found six variables with P <0.1 (**Figure 6A**). To eliminate multicollinearity in these six variables, we performed a lasso regression analysis (**Figures 6B, C****)**. Finally, four genes were acquired by multivariate Cox regression analysis, and the prediction model was developed (**Table 4**). Riskscore=WTAP*1.048+*TRMT*6*0.3159+DNMT1*-0.2019+DNMT3B*0.4305. The patients were separated into high- and low-risk groups based on their median risk score. The survival curve revealed that the high-risk group had a significantly poorer survival rate than the low-risk group (**Figure 6D**). The ROC curve of the risk score on prognosis revealed that for the first, second, and third years, the AUC was 0.716, 0.730, and 0.765, respectively (**Figure 6E**). A risk triad map was also drawn. When the risk score increases, patients’ survival time decreases, and their relative survival rate rises dramatically (**Figures 6F, G**). *WTAP*, *TRMT6*, *DNMT1*, and *DNMT3B* are distributed differently in high- and low-risk groups, as seen in the heatmap (**Figure 6H**).

**Table 4:** Multivariate Cox.

Gene	HR_95CI	P
WTAP	2.851 (2.23-3.645)	6.21E-17
TRMT6	1.371 (0.918-2.048)	0.123
DNMT1	0.817 (0.615-1.086)	0.164
DNMT3B	1.538 (1.142-2.072)	0.0004

### 3.6 Validation of CGGA

**Figure 7:**
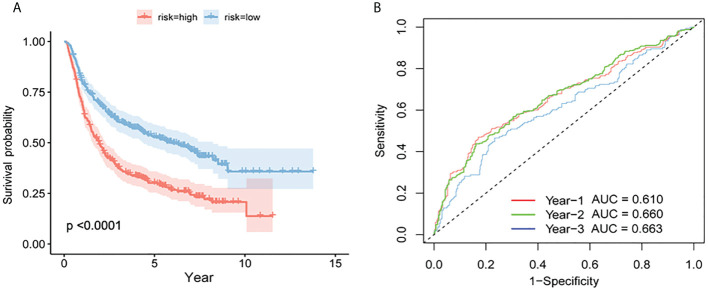
Verification of the prognostic model by CGGA. **(A)** Survival curves for high- and low-risk groups in the CGGA test set. **(B)** Test set based on time-dependent ROC curve for prognosis based on the risk score.

There were two types of patients in the CGGA database, based on the median risk score: high-risk patients and low-risk patients. High-risk patients had a poorer survival rate than low-risk patients according to the ROC curve (P = 6.32e-11, **Figure 7A**) for prognosis. The AUCs for one, two, and three years was 0.610, 0.660, and 0.663 (**Figure 7B**).

### 3.7 Immunoanalysis of high- and low-risk groups

**Figure 8:**
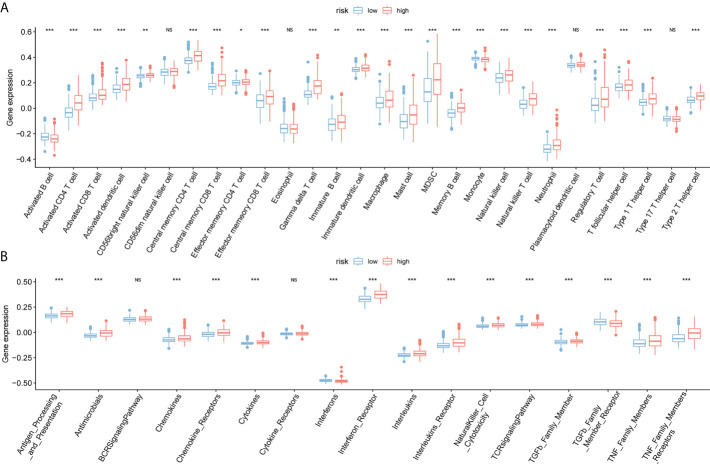
Immunoassay for ssGSEA. **(A)** The horizontal axis represents immune cells, the vertical axis represents immune cell infiltration abundance, red represents the high-risk group, and blue represents the low-risk group. **(B)** Box plot of high- and low-risk groups and immune function set scores, the horizontal axis represents the immune function set, and the vertical axis represents the immune function set score.(NS:no significant) (*P < 0.05, **P < 0.01, ***P < 0.001).

The immune infiltration comparison between high- and low-risk groups is presented in the box plot (**Figure 8A**). Among 28 types of immune cells, 24 showed significant variations in immune function sets including Antibiotics, chemokines, chemokine receptors, cytokines, interferons, interferon receptors, interleukins, leukin receptor, natural killer cell cytotoxicity, T cell receptor signaling pathway, TGFb family member, TGFb family member receptor, and tumor necrosis factor (TNF) family members. Additionally, there are notable variations in receptors between the two risk groups (**Figure 8B**).

### 3.8 Clinical correlation analysis and immunohistochemistry

**Figure 9:**
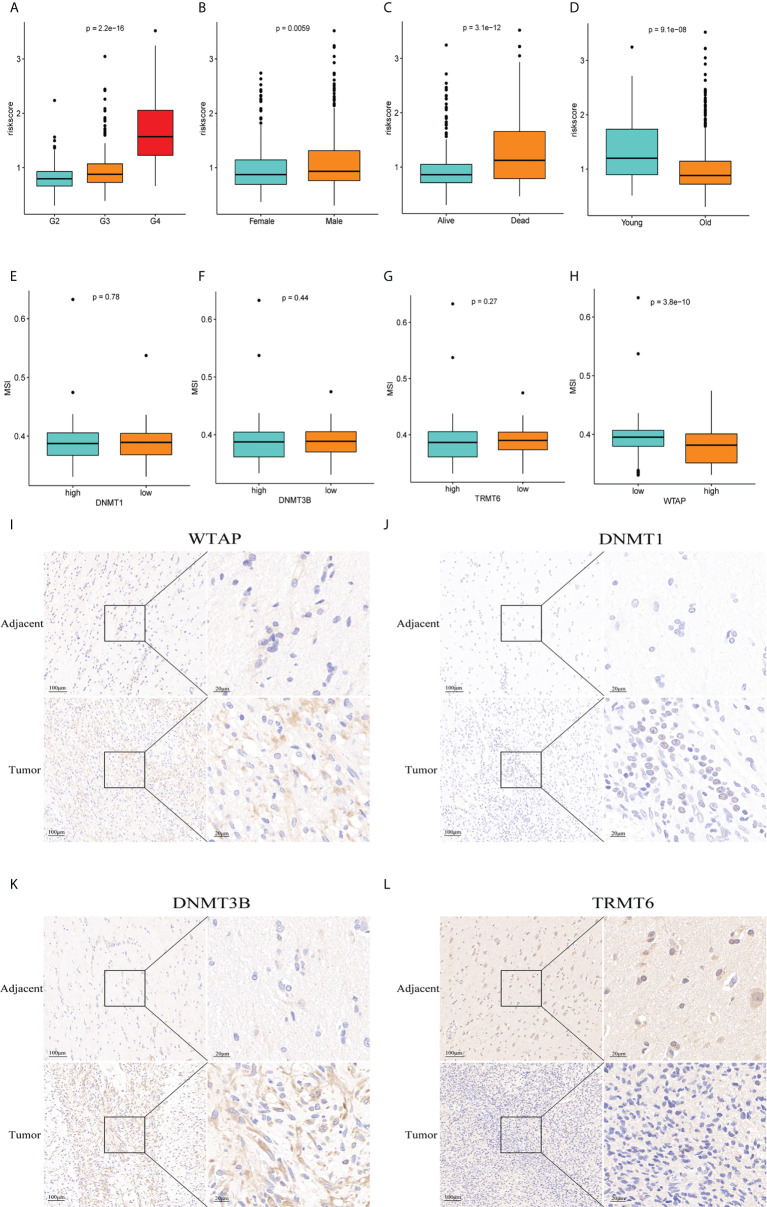
Clinical correlation analysis and immunohistochemistry. **(A–D)** There was a significant correlation between tumor grade, sex, survival status, age and risk score of patients with glioma. **(E–H)** Correlation between genes *WTA*P,*TRMT6*, *DNMT1*, *DNMT3B* and MSI (P = 0.78, P = 0.44, P = 0.27, P = 3.8e-10). Immunohistochemistry showed that the expression of WTAP **(I)**, DNMT1 **(J)**, and DNMT3B **(K)** in tumor tissue was significantly higher than that in adjacent tissue, whereas the expression of TRMT6 **(L)** was low in glioma tissue, which was consistent with the data analysis results.

In TCGA, the risk score was significantly correlated with sex, survival status, and age of the patients (p<0.05) (**Figures 9A-D**). G4 and G3 risk scores are higher than G2 risk scores, male groups are higher than female groups, death groups are higher than survival groups, and young groups are higher than old groups. We analyzed the link between *WTAP*, *TRMT6*, *DNMT1*, *DNMT3B* expression and MSI. We used the median value to divide the expression of four genes into high- and low-expression groups and investigated the relationship between genes and MSI (**Figures 9E-H**). WTAP was substantially linked with MSI, and MSI in the high-expression group was low (P = 3.8e-10). Immunohistochemistry analysis showed that the expression of WTAP (**Figure 9I**), DNMT1 (**Figure 9J**), and DNMT3B (**Figure 9K**) in tumor tissues was significantly higher than that in paracancerous tissues, whereas the expression of TRMT6 (**Figure 9L**) was low in tumor tissues. The results were consistent with the results of database bioinformatics analysis.

### 3.9 Immunity and drug resistance analysis

**Figure 10:**
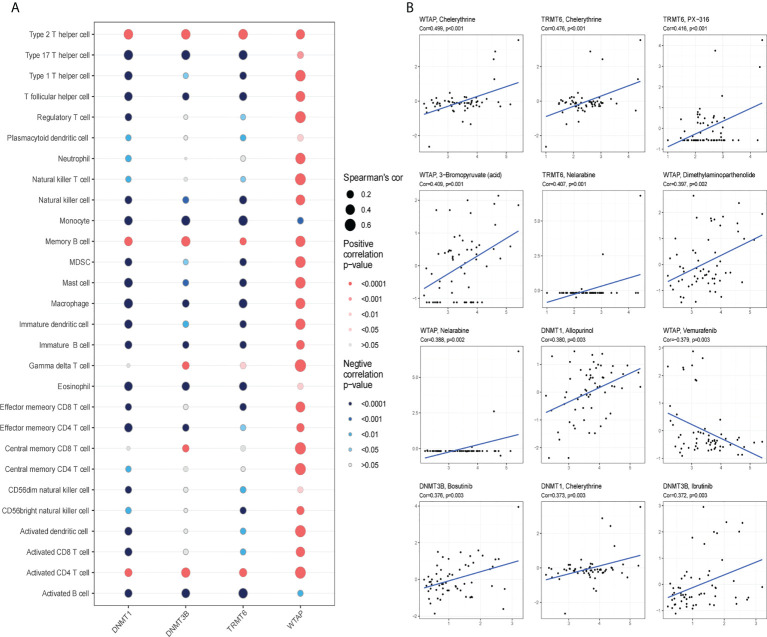
Immunoinfiltration and drug sensitivity analysis. **(A)** *WTAP*, *TRMT6*, *DNMT1* and *DNMT3B* are correlated with immune infiltration in patients with glioma. Red signifies positive correlation, blue represents negative correlation; the deeper the color is, the smaller the P-value is and the larger the dot, the larger the absolute value of the correlation coefficient. **(B)** Sensitivity of *WTAP*, *TRMT6*, *DNMT1* and *DNMT3B* to drugs (*P < 0.05).

The four hub genes had a strong association with immune cells based on gene ssGSEA data (**Figure 10A**). The genes for *TRMT6*, *DNMT1*, and *DNMT3B* had negative correlations, whereas the gene for WTAP had positive correlations with the majority of immune cells. Using the Cellminer database, the 12 most sensitive genes to medicines were ranked according to the absolute value of their correlation coefficients, and a correlation map was generated (**Figure 10B**). The expression of WTAP and the antineoplastic medication Vemurafenib were favorably connected with the sensitivity of anticancer drugs such as chelerythrine, PX-316, 3-bromopyruvate, nelarabine, and allopurinol.

## 4 Discussion

Glioma is the most common type of malignant brain tumor; especially GBM has the characteristics of invasive development, rapid progression, and a high degree of aggressiveness (22). The pathophysiology and specific mechanism of glioma are currently unknown, therapeutic options are restricted, the overall treatment impact is not optimal, and relapse after surgical resection, radiation, and chemotherapy is still common (23). Recurrent GBM has no conventional or effective treatment. A more effective treatment plan is urgently required to increase patients’ overall survival time and quality of life. Immunotherapy has shown promise in the treatment of different cancers. Researchers are currently conducting numerous studies on chimeric antigen receptor T-cell (CAR-T) immunotherapy, immune checkpoint inhibitor therapy, oncolytic virus, and tumor vaccine immunotherapy for patients with GBM with promising results, but the immune microenvironment of glioma warrants further investigation (24). The most prevalent RNA modification type at the moment is m^6^A/m^5^C/m^1^A, and there is considerable research on cancer pathogenesis (25), but limited research on glioma. Further research on the role of m^6^A/m^5^C/m^1^A-related genes in glioma and their possible implications on the tumor immune microenvironment is required.

In this study, we first analyzed the expression of 41 m^6^A/m^5^C/m^1^A-related genes in glioma and normal tissues. Twenty genes showed significant differences, including 12 m^6^A (*METTL3*, *METTL14*, *WTAP*, *VIRMA*, *RBM15B*, *FTO*, *ALKBH5*, *YTHDF1*, *HNRNPC*, *HNRNPA2B1*, *LRPPRC*, *ALKBH1*), 6 m^5^C (*NSUN2*, *DNMT1*, *DNMT3B*, *ALYREF*, *YBX1*, *TET2*), and 2 m^1^A (*TRMT6*, *RRP8*). 4 genes (*FTO*, *ALKBH5*, *LRPPRC*, *TRMT6*) were downregulated in tumor tissues, whereas the other 16 genes were upregulated in tumor tissues including *METTL3*, *METTL14*, and *NSUNA*. Upregulated expression of *METTL3* has been reported to increase resistance to temozolomide (TMZ) in patients with GBM and inhibit resensitization of drug-resistant GBM to TMZ by *METLL3* (26). Through the study of M6A of glioma stem cells, Cui et al. (27) discovered that *METTL3* and *METTL14* play an important role in the proliferation and self-renewal of glioma stem cells. Zeng et al. (28) reported that *DNMT1* is highly expressed in gliomas to promote tumor development and block tumor apoptosis *in vivo*, which is related to the WNT pathway. *NSUN2* is highly expressed in U87 and regulates the migration ability of tumor cells. Silencing *NSUN2* significantly reduces and inhibits migration (29). These results show that the differentially expressed genes do play an important role in the occurrence and development of gliomas, but there are few studies on other genes in gliomas, especially m^1^A-related genes. The GO enrichment analysis showed that the biological processes involved in differentially expressed genes mostly focused on RNA modification, and KEGG analysis revealed cancer-related pathways.

Subsequently, univariate Cox regression analysis revealed 16 differentially expressed genes with prognostic values, 2 clusters were detected by consistent cluster analysis, and 10 genes were differentially expressed between cluster 1 and cluster 2. The overall survival rate of patients with glioma in cluster 2 was higher than that of those in cluster 1. Cluster 1 was investigated using GSEA to discover its biological roles, which included cell cycle, immunological microenvironment, and receptor modulation. The pathway enriched by cluster 2 has been linked to various glioma oncogenic pathways (30, 31). We also analyzed the immune cell infiltration score and the immune function gene set in clusters 1 and 2. Except for activated B cells and monocytes, there were statistical differences between 26 types of cells in clusters 1 and 2, and the infiltration level of most immune cells in cluster 1 was higher than in cluster 2. Hara et al. (32) discovered that macrophages directly induced GBM cells to change into a mesenchymal-like state, which was linked to an increase in the abundance and cytotoxicity of tumor-infiltrating T cells, implying a functional interaction between immune cells and the GBM cell state. Friedrich et al. (33) discovered that glioma cells could penetrate immune cells by reprogramming some common mutations, effectively crippling the human immune system’s fight against the brain. To that aim, the researchers have devised a new therapeutic that reactivates the “paralyzed” immune system in mice with isocitrate dehydrogenase mutant tumors, allowing them to live longer. There were significant differences in the scores of 17 immune function gene sets between the two clusters. A recent study (34) has reported that it is not the cancer cells that consume a considerable amount of glucose. The researchers used 18F-FDG-PET to detect glucose consumption in mouse tumor models to quantify glucose intake by different cell populations in the tumor microenvironment. Glucose intake by infiltrating immune cells was larger than that by cancer cells. Our findings strongly imply that immunotherapy may have a curative effect on glioma patients, but further research is needed.

Then, utilizing m^6^A/m^5^C/m^1^A-related genes, we created a gene prediction model that includes *WTAP*, *TRMT6*, *DNMT1*, and *DNMT3B*. The median risk score was separated into two groups: high-risk and low-risk. The high-risk group’s survival time was dramatically reduced, and the calculated risk score performed well in predicting the prognosis of the patients with glioma. We further tested the model’s prediction efficiency and reliability using the CGGA database, which contains 693 glioma patients. *WTAP* was found to be significantly expressed in glioma tissues among the four genes studied, and its high expression was linked to poor postoperative survival (35). *WTAP* can also encourage glioma cell invasion and migration (36, 37). *DNMT1* plays a role in glioma growth, apoptosis, and migration (28). Glioma growth is slowed *in vitro* and *in vivo* experiments when a *DNMT1* inhibitor is given (36). Currently, there is no specific research on *DNMT3B* in glioma, but *DNMT3B* has been reported to accelerate the occurrence and progression of esophageal cancer (38), lung cancer (39), breast cancer (40), and ovarian cancer (41), implying that *DNMT3B* is an essential biomarker in cancer pathogenesis.

Immune cell analysis and immune function set analysis revealed valuable results in this study. 24 types of immune cells in high- and low-risk scores were significantly different, and *TRMT6*, *DNMT1*, and *DNMT3B* were mainly negatively correlated with immune cells, whereas *WTAP* was positively correlated with most immune cells. TGFb family members, TNF family members, and TNF family member receptors were among the 15 immune function sets with significant variations. Blocking *DNMT1* can stop TGF-induced glioma cell growth, migration, and invasion (42). By increasing the expression of *TNFAIP3*, the *Circ0008399*/*WTAP* combination can prevent bladder cancer cells from dying. The *circ0008399*/*WTAP*/*TNFAIP3* pathway can help increase cisplatin treatment sensitivity in bladder cancer (43). This research supports the scientific validity of our findings. The risk of death in the G4 and G3 groups was more than twice as high as that of survival in the G2 group, and in the male group, it was more than twice as high as that in the female group. The risk of death was also more than double that of survival. Only *WTAP* was significantly correlated with MSI, with MSI in the high expression group being relatively low. Finally, sensitivity analysis of antineoplastic drugs showed that except for the negative correlation between *WTAP* expression and antineoplastic drug Vemurafenib, there was a positive correlation between gene expression and anticancer drug sensitivity, indicating that the high expression of genes is more likely to help patients benefit from antineoplastic drugs. Park et al. (44) reported that *DNMT1* levels can alter the susceptibility of patients with glioma to Decitabine, and *DNMT1* can be used to predict glioma responsiveness to Decitabine therapy. Decitabine can help patients with glioma who have high *DNMT1* expression. Zhou et al. (45) showed that *DNMT1* mediates chemosensitivity by reducing the methylation of *microRNA-20a* promoter in glioma cells, and the expression of *DNMT1* in drug-resistant U251 cells is downregulated. The epigenetic modulation of TMZ during chemotherapy in patients with GBM is not yet known. Undoubtedly, there are some limitations to our study. The mechanism of how the identified 4 genes participate in the regulation of the tumor immune microenvironment is unclear. The prognostic model needs to be verified in a large-scale and multicenter clinical cohort. However, this study does provide a comprehensive overview of m^6^A/m^5^C/m^1^A-related genes in glioma, which guide us to further study the role of RNA methylation modification in glioma in the future.

## 5 Conclusion

We systematically assessed the expression of m^6^A/m^5^C/m^1^A-related genes in glioma, identified different clusters of m^6^A/m^5^C/m^1^A-related genes using consistent cluster analysis in glioma, and investigated the potential biological function mechanism of clusters 1 and 2 as well as the role of immune cells and immune functions. We created a four-gene prognostic marker, and a validation model is a viable tool for predicting the survival outcomes of patients with glioma. Compared with the paracancerous tissues, the immunohistochemical results showed that the expression of TRMT6 was low in the tumor, and the other three genes were significantly higher, which was consistent with the results of the bioinformatics analysis. Finally, the investigation of anti-tumor drug sensitivity, immunological microenvironment, and clinical features yielded valuable results. These important findings will serve as the foundation for additional research into the prevalence, development, and impact of m^6^A/m^5^C/m^1^A-related genes in glioma.

## Data availability statement

The datasets presented in this study can be found in online repositories. The names of the repository/repositories and accession number(s) can be found in the article/**Supplementary Material**.

## Ethics statement

The studies involving human participants were reviewed and approved by the Clinical Ethics Committee of the Second Affiliated Hospital of Kunming Medical University. The patients/participants provided their written informed consent to participate in this study.

## Author contributions

Data curation: KaZ, WL. Methodology: MH, JL, YD. Writing - original draft: XH, KuZ. Writing-review and editing: NZ, YY. All authors contributed to the article and approved the submitted version.

## Funding

This study was supported by the National Natural Science Foundation of China (Grant No.82172998) and the Project of Yunnan Provincial Department of Science and Technology(No.202201AY070001-098).

## Acknowledgments

The authors thank all the staff of the Department of Neurology, the Second Affiliated Hospital of Kunming Medical University.

## Conflict of interest

The authors declare that the research was conducted in the absence of any commercial or financial relationships that could be construed as a potential conflict of interest.

The reviewer CL declared a shared affiliation with the authors to the handling editor at time of review.

## Publisher’s note

All claims expressed in this article are solely those of the authors and do not necessarily represent those of their affiliated organizations, or those of the publisher, the editors and the reviewers. Any product that may be evaluated in this article, or claim that may be made by its manufacturer, is not guaranteed or endorsed by the publisher.
